# Cracking the Code: The Impact of Orthographic Transparency and Morphological-Syllabic Complexity on Reading and Developmental Dyslexia

**DOI:** 10.3389/fpsyg.2018.02534

**Published:** 2019-01-04

**Authors:** Elisabeth Borleffs, Ben A. M. Maassen, Heikki Lyytinen, Frans Zwarts

**Affiliations:** ^1^Center for Language and Cognition Groningen, School of Behavioural and Cognitive Neurosciences, University of Groningen, Groningen, Netherlands; ^2^Department of Psychology, Agora Center, University of Jyväskylä, Jyväskylä, Finland; ^3^Niilo Mäki Institute, Jyväskylä, Finland

**Keywords:** orthographic transparency, syllabic complexity, morphological complexity, dyslexia, reading models

## Abstract

Reading is an essential skill in modern societies, yet not all learners necessarily become proficient readers. Theoretical concepts (e.g., the orthographic depth hypothesis; the grain size theory) as well as empirical evidence suggest that certain orthographies are easier to learn than others. The present paper reviews the literature on orthographic transparency, morphological complexity, and syllabic complexity of alphabetic languages. These notions are elaborated to show that differences in reading acquisition reflect fundamental differences in the nature of the phonological recoding and reading strategies developing in response to the specific orthography to be learned. The present paper provides a narrative, cross-linguistic and integrated literature review, thereby contributing to the development of universal reading models and at the same time pointing out the important differences between orthographies at the more detailed level. Our review also yields suggestions to devise language-specific instruction and interventions for the development of the specific reading strategies required by the characteristics of the orthography being acquired.

## Introduction

Reading is an essential skill in modern societies, yet depending on the orthography and exact diagnostic criteria used, 5–17.5% of children suffer from dyslexia and face persisting problems with reading and spelling (Habib and Giraud, 2013). During the past decade, much progress has been made in our understanding and treatment of dyslexia (Shaywitz et al., 2008; Lyytinen et al., 2009; Van der Leij et al., 2013; Peterson and Pennington, 2015). Furthermore, research no longer focuses solely on English; reading problems in other languages have been receiving increased attention (Landerl et al., 2013; Peterson and Pennington, 2015). Nonetheless, the mechanisms of typical reading acquisition and the causes of reading deficits remain complex, which makes it all the more fascinating that after deliberate practice, a process this intricate comes so naturally to many of us despite differences in socio-economic backgrounds, intellectual capacities, and the characteristics of the language script being learned.

The beginning reader of any alphabetic language basically needs to learn to associate letters with sounds in order to access whole-word phonological representations of known words (Grainger and Ziegler, 2011). At first, reading will involve a serial letter-by-letter strategy, as the mechanism for parallel letter identification has not yet been established. The different letters of the word are identified one at a time by shifts of the eyes and shifts of attention while the reader learns what sounds they correspond to. This process hinges on two key sources of information available at this point: alphabetical knowledge and spoken vocabulary (Grainger and Ziegler, 2011).

Thanks to their ability to sound out words, readers can establish high-quality lexical representations with multiple redundant connections between phonological and orthographic representations of words in their memory, also including morphological and semantic knowledge. Ultimately, once lexical representations have been established, the reader no longer needs to rely on phonics when encountering the same word again, which increases the speed and efficiency of the reading process. The ability to translate written text to spoken words with speed and accuracy is also denoted as oral reading fluency and is considered an overall indicator of reading development and competence (Fuchs et al., 2001). In their model of information processing in reading, LaBerge and Samuels (1974) postulate that skilled reading involves the redistribution of the reader’s attentional capacity from lower level word identification procedures to high-level resource-demanding comprehension processes (Fuchs et al., 2001). LaBerge and Samuels (1974) describe how the execution of a complex skill requires the coordination of many component processes within a very short time frame. If each component requires attention, the attentional capacity would be exceeded, making performance of the complex skill impossible. If, however, enough components can be processed automatically, the attentional load stays within tolerable limits, allowing for successful performance. One of the first and vital tasks for beginning readers hence is to develop rapid and automatic word-recognition procedures (Sprenger-Charolles and Colé, 2003) in order to become a fluent reader.

The ease with which a new letter string can be translated into a phonological code will depend to a large extent on how easily the letters of new words map onto the sounds of the corresponding spoken words. The specific orthography learned has been put forward as a central environmental factor influencing reading acquisition and dyslexia (for a review, see Ziegler and Goswami, 2005). Moreover, orthographic differences across languages have been shown to impose differential weighting on neural pathways during word-reading (e.g., Das et al., 2011). With reading research suggesting that certain orthographies are easier to learn than others (e.g., Aro and Wimmer, 2003; Seymour et al., 2003), one is curious to know which orthographic components of a language have been identified as causing these differences in complexity. Furthermore, like us, many researchers question whether these differences indeed affect the development and expression of dyslexia, and if so, in what way. Recently, Borleffs et al. (2017) published a paper discussing quantitative metrics measuring differences between alphabetic languages in orthographic transparency, morphological complexity, and syllabic complexity. According to the authors, more research is needed to understand the ‘developmental footprint’ of these variables in the lexical organization and processing strategies being developed for reading. The current paper therefore reviews the literature on orthographic transparency, morphological complexity, and syllabic complexity of alphabetic languages trying to provide more insight into their influence on reading acquisition and developmental dyslexia. This narrative review thereby focuses on the implications for theory development and modeling, aiming to learn from linguistic analyses of alphabetic scripts to better understand the neurocognitive processes involved in impaired and unimpaired reading.

Our review of the literature included searches of PubMed, PsycInfo, Web of Science, and Google Scholar. The search terms used pertained to orthographic transparency, morphological complexity, and syllabic complexity in relation to research on orthographic differences, reading models, reading acquisition, and developmental dyslexia. Only studies written in English related to the abovementioned topics were included in this review, without limiting the search results based on the year of publication. This led to the inclusion of a total of 159 papers. During the initial literature searches, we observed that to date, little research has focused on whether other orthographic properties besides orthographic transparency have an impact on reading acquisition and developmental dyslexia. This has motivated our decision to additionally include the impact of syllabic and morphological complexity of the specific orthography to be acquired. Our goal was to include as many papers as possible within the boundaries of the scope of the paper, with the aim to create a more complete and detailed understanding of the various factors that determine a language’s complexity.

## Orthographic Transparency

Complex and opaque orthographic mapping systems can cause particular problems, not only to children having to cope with dyslexia (Landerl et al., 1997). Even though all orthographies describe the sound structure they represent, there is considerable variability in how transparent this grapheme-phoneme relationship is to the learner. This variability in orthographic depth (transparency, regularity, consistency) is caused by differences in the degree of systematicity with which letter sequences map onto their corresponding phoneme sequences (e.g., Aro, 2004; Protopapas and Vlahou, 2009; Ziegler et al., 2010; Caravolas et al., 2012; Landerl et al., 2013). In languages with a transparent mapping system, such as Indonesian, Italian, or Finnish, surface phonology is reflected in spelling with a high level of consistency. In those languages, the pronunciation of a given letter of the alphabet is almost always the same irrespective of the word they appear in Aro (2004), Ziegler et al. (2010), and Winskel and Lee (2013). In opaque orthographies, such as Danish and English, however, orthography-phonology relationships can be very ambiguous (Seymour et al., 2003; Frost, 2012). In English, generally considered the least consistent among Indo-European languages (e.g., Seymour et al., 2003; Share, 2008; Frost, 2012), a given letter is often pronounced differently in different words like the ‘a’ in *bag, lake, was*, and *raw*. Some letters have no corresponding sound (e.g., ‘w’ in *answer*), while the same sound can have multiple spellings (e.g., /k/ in *calm, king, opaque*, and *track*). Consequently, many English words cannot be sounded out accurately if the word is not part of the reader’s vocabulary. Morphological variations in English are characterized by an extensive amount of phonological variations. Changes in pronunciation (e.g., *courage*-*courageous, heal*-*health*) due to derivations, inflections, addition of suffixes, changes in stress due to affixation, and so on, have caused the English orthography to evolve into a highly inconsistent writing system (Frost, 2012). In French, the pronunciation in some cases depends on the context; the numeral *dix* is for example pronounced as /di/ in *dix voitures* (‘ten cars’), /diz/ in *dix arbres* (‘ten trees’), and /dis/ in *tu as dix* (‘you have ten’) (Carrillo et al., 2013). It seems obvious that these factors will considerably complicate the reader’s decoding task and that positional restrictions of some spelling-to-sound combinations (context dependency) need to be part of a reader’s knowledge (Borgwaldt et al., 2005).

Orthographic transparency manifests itself in a feedforward fashion (grapheme-to-phoneme) and a feedback fashion (phoneme-to-grapheme) (Lété et al., 2008). The English orthographic system is regarded symmetrical, with its orthography being irregular in both directions. Although Finnish grapheme-phoneme correspondences are also symmetrical, they are, in contrast to English, regular in both directions. Some writing systems are irregular in one direction only. French and German, for instance, are regarded as relatively regular from the reading point of view but less so from a spelling perspective (Aro, 2004).

In addition to the potential difficulties arising from the complexity of grapheme-phoneme relationships, another component of orthographic transparency concerns the complexity of determining the graphemic elements of a word (graphemic parsing). Languages differ with respect to possible and typical graphemes (single letters or letter clusters) which are governed by language-specific graphotactic, syllabic, and morphological constraints. Thus, to be able to transform the four-letter string of the French word *chat* (‘cat’) into the two-phoneme translation /∫ α/, the reader first has to be aware that the string *chat* contains three graphemes ‘ch,’ ‘a,’ and ‘t,’ and, secondly, that ‘ch’ maps onto /∫ / and ‘at’ onto /α/ in this particular context. This requires knowledge of which letter clusters can occur in the French orthography and which possible correspondences there are between graphemes and phonemes in this language (Van den Bosch et al., 1994).

Theoretically, the evolution of writing systems could have closely followed the phonological forms of a language and conveyed the different pronunciations of different morphological variations to the reader. However, several writing systems have evolved to provide readers with the meaning of the printed forms by indicating their etymological and morphological origins rather than by simplifying phonological decoding. In these orthographies, the level of orthographic transparency has essentially been influenced by morphological information, thereby modulating the complexity of the reading process. As a consequence, knowledge of grapheme-phoneme correspondences alone will not suffice to decide on the correct spelling, pronunciation, and meaning in every language.

### Orthographic Transparency and Reading Acquisition

Several researchers have raised the important question as to what extent the cognitive mechanisms underlying reading acquisition vary across orthographies. Theoretical considerations (e.g., the orthographic depth hypothesis (ODH) by Katz and Frost, 1992, and the grain size theory by Ziegler and Goswami, 2005) as well as empirical evidence (e.g., Aro and Wimmer, 2003; Seymour et al., 2003; Caravolas et al., 2013) suggest that transparent orthographies with highly regular grapheme-phoneme mappings are acquired more easily than opaque and complex orthographies with a high proportion of inconsistent and irregular spellings.

The majority of the English-based reading acquisition models share a common idea of dual-processing routes. The traditional ‘dual-route’ models (Coltheart et al., 1993, 2001; Perry et al., 2007, 2010) conceive of a direct, lexical route for whole-word recognition and an indirect, sublexical route for phonological decoding, suggesting that readers adapt their reliance on the two processing routes depending on the demands of the orthography.

In line with this notion that during learning the reading process is adapted to the orthography being decoded, the ODH proposes that word identification in shallow orthographies is primarily based on phonological pre-lexical computation, whereas in deep orthographies this process relies strongly on orthographic cues (Katz and Frost, 1992; Frost, 2005). This ‘strong’ ODH, which labels the core processes as either ‘phonological’ or ‘orthographic’ according to the depth of an orthographic system, also has a ‘lighter’ version in which orthographic knowledge is assumed to be involved in word identification in shallow orthographies (Carrillo et al., 2013). Nonetheless, even in this less stringent model, the role of phonology is considered far more important in shallow than in deep orthographies.

Several monolingual studies of reading in relatively transparent writing systems have found high accuracy scores for word and pseudoword decoding toward the end of the 1st year of formal education. Cossu et al. (1995), for example, showed that Italian children were, on average, able to read 94% of the presented words and 82% of the pseudowords correctly, while Greek children were shown to have attained an average accuracy level of 90% for words and 89% for pseudowords by the end of the first grade (Porpodas et al., 1990). This rapid development of decoding skills in transparent orthographies is attributed to straightforward grapheme-phoneme conversion rules that do not place high demands on phonological processing for decoding (Aro et al., 1999; Mann and Wimmer, 2002). By contrast, English-speaking children have been found to perform relatively poorly on accuracy tests: by the end of the 1st year, Scottish children were able to read 29% of English pseudowords correctly (Seymour et al., 2003).

Assessing numeral reading, number-word reading, and pseudoword reading in their cross-linguistic study, Aro and Wimmer (2003) compared the performance of English-speaking children in grades 1 to 4 with that of same-year children speaking German, Dutch, Finnish, French, Spanish, and Swedish. By the end of the 1st year, reading accuracy for pseudowords was already around 85% for the Dutch, German, Finnish, and Spanish children and over 90% for the Swedish-speaking children, while the English-speaking children had achieved a 50% accuracy only; they did not reach their peers’ high accuracy levels until grade 4. Based on these results, the authors concluded that the ability to translate unknown letter strings into acceptable pronunciations was easily attained in all orthographies studied, except for English.

Seymour et al. (2003) demonstrated the impact of orthographic complexity on reading development by evaluating 13 European orthographies. The authors placed English at the far end of their classification as possessing the most complex orthographic system of all European languages included. In the majority of countries, children were able to read familiar words and had attained simple decoding skills before the end of the 1st year, while readers acquiring deeper orthographies (French, Portuguese, Danish, and English) were still struggling. Their results suggested that the speed of early reading acquisition in English was slower by a ratio of as much as 2.5:1 compared to most European orthographies. These results are in accordance with other studies confirming that learning to read is easier in more shallow orthographies. They include comparisons of Dutch with English (Patel et al., 2004), German with English (Wimmer and Goswami, 1994), English with Turkish (Öney and Goldman, 1984), Spanish with French, and English (Goswami et al., 1998), English with Welsh (Spencer and Hanley, 2003), Hungarian, Portuguese, Dutch, French, and English (Ziegler et al., 2010), and English with Spanish, and Czech (Caravolas et al., 2013).

According to Seymour et al. (2003), the delayed acquisition of foundation literacy in English and to some extent also in Danish, can be interpreted as a combined effect of an inconsistent system of grapheme-phoneme mappings and a complex syllabic structure. The authors define this complex syllable structure in terms of the presence of numerous initial and final consonant clusters and a predominance of closed CVC syllables. Syllabic complexity will be discussed more extensively later in this paper. Seymour and colleagues hypothesize that, in accordance with ODH, in the deeper orthographies reading acquisition may be based on the formation of a dual (a logographic and an alphabetic) foundation, which takes more than twice as long to attain as the single (alphabetic) process when learning a shallow orthography. Within this foundation-literacy framework (Seymour, 1990, 1997, 1999), the logographic process facilitates the identification and storage of familiar words, whereas the alphabetic process enables sequential decoding. The authors propose that dual-process learning, which implies the concurrent development of both processing routes, demands the involvement of a wider range of cognitive skills than single-process learning. Arguably, in conditions in which attention and processing resources need to be divided between learning processes, learning will occur more slowly than it will under conditions in which all resources are dedicated to a single process. The authors suggest that there is a threshold of orthographic complexity which, once exceeded, drastically changes the cognitive architecture of the reading process with the introduction of a dual-process system. If, however, the orthography meets the relevant criteria of simplicity, then a single-process alphabetic foundation will be developed as the basis for later reading. Following their comparison of reading acquisition across European orthographies, the authors propose that Portuguese, French, Danish, and English exceed the threshold while the other nine orthographies (Dutch, German, Greek, Finnish, Norwegian, Spanish, Icelandic, Swedish, and Italian) do not.

Along similar lines, in their grain size theory Ziegler and Goswami (2005) argue that the differences in reading acquisition as shown by Seymour et al. (2003) study reflect fundamental differences in the nature of the phonological recoding and reading strategies developing in response to the specific orthography to be learned. Unlike Seymour at al., however, their framework focuses on the different sizes of the orthographic units (i.e., grain size) the reader uses rather than two separate processing routes. The authors hypothesize that children who are trying to master an orthographically more consistent alphabetic language such as Greek, Italian, German, or Spanish rely heavily on grapheme-phoneme recoding strategies as these mappings are relatively consistent, whereas children learning to read less consistent orthographies cannot use smaller grapheme units as easily because, at least in English, smaller grain sizes tend to be less consistent than larger grain sizes (Treiman et al., 1995). This may well result in the development of recoding strategies that facilitate decoding at the level of multiple grain sizes, complementing grapheme-phoneme conversion strategies with the recognition of letter patterns for rimes^1^ and attempts at whole-word recognition. Indeed, English-speaking children have been found to benefit from a focus on larger units such as rimes as part of reading instruction (Kyle et al., 2013).

Seymour et al. (2003) and Ziegler and Goswami (2005) all question the extent to which the two processing routes proposed in the dual-route approach develop in more transparent orthographies. Assuming the extreme position of English with regard to spelling-sound relationships, Ziegler and Goswami even argue that some of the most refined processing architectures, such as two separate routes to pronunciation in the skilled reading system, may in fact only develop in speakers of English. Similar to Ziegler and Goswami (2005), a growing number of researchers have raised doubts about the generalizability of the dual-route system beyond English (e.g., Hutzler and Wimmer, 2004; Share, 2008), and have criticized the “Anglocentrism” (Share, 2008) in reading research. One may indeed argue that if the orthography-phonology relationships are regular, then a second, lexical route tailored specifically to whole-word recognition would be dispensable. In that case, a more parsimonious one-route model would suffice to be able to read every pronounceable word and pseudoword.

Share (2008), on the other hand, observes that the aforementioned reservations about the existence of a lexical route for both opaque and transparent orthographies ironically overlook a fundamental duality in reading that involves all words in every possible orthography, irrespective of their level of (ir)regularity. Every word is visually unfamiliar at some point in reading development, which implies that every new reader must possess some kind of strategy to individually identify words he or she encounters for the first time. Moreover, eventually, every reader must attain a high level of automatization in visual word recognition to rapidly and effortlessly recognize familiar morphemes and words (Perfetti, 1985) that are then processed as whole units by a direct-retrieval mechanism (Ans et al., 1998). Share (2008) argues that the ability to automatize or modularize the word-identification process is probably the essence of the reading skill (Perfetti, 1985), even more so than the ability to build the accurate pronunciation of words varying in spelling-sound consistency. Hence Share (2008) claims that both the decoding strategy and this rapid, direct-retrieval mechanism apply to all words in all orthographies, both exceptional and regular.

In line with this claim, Caravolas et al. (2013) argue that based on the results of a longitudinal study of the development of early literacy skills in English, Spanish, and Czech, differences in orthographic depth will not demand for the involvement of different cognitive mechanisms but will mainly be expressed in the rate of reading development. Although the growth in reading skills in Caravolas et al.’s (2013) study was indeed found to be slower and followed a different trajectory in English than in the more consistent Czech and Spanish orthographies, the similar patterns of prediction of variations in reading development from three core cognitive skills, i.e., phoneme awareness, rapid automatized naming, and letter knowledge, suggest that the same cognitive mechanisms underlying reading acquisition are involved across the different languages (also see Vaessen et al., 2010).

### Orthographic Transparency and Dyslexia

Although dyslexia has been studied intensively over the past decades, no scientific consensus has been reached yet about the underlying cognitive and biological causes of this developmental condition (see e.g., Wolf and Bowers, 1999; Ramus et al., 2003; Pennington, 2006). One major theory of developmental dyslexia is the phonological theory, which postulates that developmental dyslexia originates from specific impairments in the representation, storage and/or retrieval of speech sounds (Shaywitz, 1998; Ramus et al., 2003). Other theories, however, such as the magnocellular theory (Stein and Walsh, 1997) and the cerebellar theory (Nicolson and Fawcett, 1990) have also found empirical support (e.g., Livingstone et al., 1991; Fawcett et al., 1996; Leonard et al., 2001; Van Ingelghem et al., 2001).

Ziegler and Goswami (2005) argue that children with dyslexia in different languages exhibit common phonological deficits and that predictors of reading performance, at least in alphabetic languages, are relatively universal. Nonetheless, the predictors’ precise weights may vary as a function of the transparency of the mapping system, as might be true for the other indicators of developmental dyslexia. In more transparent languages, rapid automatized naming has been claimed to be a stronger predictor of reading skills than phonological awareness (e.g., De Jong and Van der Leij, 1999, 2003; Wimmer et al., 2000) and in contrast to phonological awareness, the relative importance of rapid automatized naming has been shown to increase over time (De Jong and Van der Leij, 1999; Vaessen et al., 2010; Heikkilä et al., 2016). Conflicting results, however, have also been reported regarding the relationship between both predictors and reading (e.g., Landerl et al., 2013; Moll et al., 2014) and those relationships hence warrant more research. It should be noted that predictors of reading skills and dyslexia, as well as the abovementioned theories of dyslexia, will not be further discussed in this review. We acknowledge that there is a substantial body of research addressing these topics, such as the specific role RAN plays in the development of reading fluency in dyslexic readers (see e.g., Jones et al., 2009, 2013, 2016) but this falls outside the scope of the present review.

Research has shown that reading speed is usually slowed in children with reading difficulties in transparent orthographies. Reading accuracy, however, remains more or less unaffected after the very early phases of reading acquisition (e.g., Holopainen et al., 2001; Tressoldi et al., 2001; Landerl and Wimmer, 2008; Constantinidou and Stainthorp, 2009; Dandache et al., 2014) and their phonological decoding and phoneme identification hence seems to be relatively intact (Ziegler and Goswami, 2005; Barca et al., 2006; Martens and De Jong, 2006). If grapheme-phoneme relationships are consistent, even dyslexic children are apparently able to map written words onto their spoken forms. It has even been suggested that in languages with a transparent orthography (German in this case) symptoms of poor reading are less severe in children at the lower end of the reading ability distribution than they are in their English counterparts, at least in terms of accuracy (Landerl et al., 1997). Results from the same studies also indicated, however, that in transparent orthographies the rate of word decoding in poor readers was relatively low compared to that of typical readers, while it should likewise be noted that several other studies found some of the poor readers in transparent orthographies to demonstrate a tendency toward inaccurate reading as well (e.g., Sprenger-Charolles et al., 2000; Boets et al., 2010; Eklund et al., 2015).

A study of diagnostic profiles of dyslexic Dutch-speaking children by Tilanus et al. (2013) suggests that in a transparent orthography reading is a matter of speed rather than accuracy (also see Landerl and Wimmer, 2008), and that it is a phonological deficit that underlies the reading problems (Wimmer and Schurz, 2010; Verhoeven and Keuning, 2018). To explain the low reading speed, it is proposed that dyslexic readers persevere in using an inefficient, sublexical decoding strategy (De Luca et al., 2002; Hutzler and Wimmer, 2004; Spinelli et al., 2005; Martens and De Jong, 2006) instead of developing a reliance on a more efficient parallel word-recognition strategy, as is the case in typical reading development (Tilanus et al., 2013). This notion would coincide with findings that in dyslexic readers reading speed (Spinelli et al., 2005; Zoccolotti et al., 2005; Martens and De Jong, 2006; Marinus and De Jong, 2010) and accuracy (Verhoeven and Keuning, 2018) are affected by word length.

Assuming lexical and sublexical processing routes for reading, models of impaired reading acquisition have been developed describing different types of reading difficulties depending on the component of the skill being most affected. In these models of impaired reading acquisition, notions from acquired dyslexia such as ‘surface dyslexia’ (disordered word-specific lexical processing) and ‘phonological dyslexia’ (disordered grapheme-to-phoneme conversions) have been shown to also apply to developmental dyslexia (e.g., Ellis, 1985; Aro, 2004; Peterson et al., 2013, 2014).

In their review of the prevalence and reliability of dyslexic subtypes in languages varying in orthographic depth (French, Spanish, and English), Sprenger-Charolles et al. (2011) conclude that comparisons of dyslexic children and reading level controls reveal that the ‘phonological subtype’ of developmental dyslexia corresponds to a *deviant* developmental trajectory across all three languages, whereas the ‘surface subtype’ reflects a *delayed* development. Except in the study by Jiménez et al. (2009), the orthographic skills of the children with surface dyslexia did not differ from those of the reading-level controls in the articles they evaluated (Castles and Coltheart, 1993; Manis et al., 1996; Stanovich et al., 1997; Génard et al., 1998; Sprenger-Charolles et al., 2000; Ziegler et al., 2008; Jiménez et al., 2009). It is worth noting that the orthographic deficit observed in the Jiménez et al. (2009) study was found to be related to poor home literacy experiences (e.g., print exposure). By contrast, the phonological reading abilities of the phonological dyslexics were systematically inferior to those of the controls (Manis et al., 1996; Stanovich et al., 1997; Sprenger-Charolles et al., 2000), and some studies observed the same effects in the phonological reading abilities of surface dyslexics (e.g., Sprenger-Charolles et al., 2000). Moreover, in studies assessing phonemic awareness (Manis et al., 1996; Stanovich et al., 1997; Sprenger-Charolles et al., 2000; Ziegler et al., 2008; Jiménez et al., 2009) the researchers found a phonological deficit in the phonological dyslexics, while three studies also uncovered this deficit in surface dyslexics (Sprenger-Charolles et al., 2000; Ziegler et al., 2008; Jiménez et al., 2009). For this finding, Sprenger-Charolles et al. (2011) offered the explanation that the surface-dyslexic profile may develop from a mild phonological deficit together with a lack of reading opportunities. This would explain why surface dyslexics are frequently found to have both impaired phonological and orthographic reading abilities, the latter impairment being explained by the fact that the establishment of well-defined orthographic representations requires frequent exposure to print (Harm and Seidenberg, 1999). The Sprenger-Charolles et al. (2000) review furthermore showed that cross-language differences were noticeable in the distribution of dyslexic profiles. When the classification was based on accuracy scores, the percentages of surface dyslexics were higher than those for phonological dyslexics in the Spanish and French studies (Génard et al., 1998; Sprenger-Charolles et al., 2000; Ziegler et al., 2008; Jiménez et al., 2009), but no such systematic difference was obtained in the accuracy-based English studies (Casalis et al., 2004; Manis et al., 1996; Stanovich et al., 1997). According to Sprenger-Charolles et al. (2011) these results do not indicate that phonological decoding deficits are non-existent in transparent orthographies but rather that reading speed needs to be considered to detect such a deficit (also see Share, 2008).

Schiff et al. (2013) explored the effects of orthographic transparency on the reading development of fourth-grade readers of Hebrew, revealing a different developmental pattern among the children with dyslexia. The Hebrew script is characterized by a special denotation of vowel information and consists of both a vowelized and an unvowelized script. Whereas the vowelized Hebrew script is regarded highly consistent and regular representing both consonants and vowels using vowel letters as well as diacritic marks, the unvowelized script is considered orthographically inconsistent and irregular as it does not include any diacritics to represent vowels that are not conveyed by the basic alphabet (Schiff, 2012). Interestingly, their results suggested that, in contrast to typically developing young readers of Hebrew who were found to rely on vowelization for the acquisition of orthographic representations during the early stages of reading, no such reliance was found among the dyslexic readers. The authors propose that this might be the result of the dyslexic readers’ flawed grapheme-phoneme conversions skills, impeding the use of the vowelized script as a self-teaching mechanism for the development of an orthographic lexicon needed for the later decoding of unvowelized words (Share, 1995). As no significant differences were found in reading accuracy between the consistent vowelized and the inconsistent unvowelized scripts, vowelization was not shown to contribute to reading accuracy among the dyslexic children. These findings are at odds with previous cross-linguistic studies showing that dyslexic children reading transparent orthographies performed better on reading accuracy tasks than those having to master more opaque orthographies (Landerl et al., 1997; Ziegler et al., 2003). Schiff et al. (2013) propose that dyslexic children in languages with both vowelized and unvowelized scripts possibly minimize the role of vowelization in phonological decoding and might perceive the different scripts within the language as being similar. This would in turn indicate that the severe difficulties that these dyslexic children are experiencing in Hebrew might prevent them from developing more efficient reading strategies.

## Morphological Complexity

A significant amount of words we read every day are morphologically complex. In French and English, for example,this concerns approximately 75 and 85% of the words, respectively (Grainger and Ziegler, 2011). Morphologically complex words such as *work* may, for example, have inflected forms (e.g., *works, workers, working*), prefixed and suffixed derivations (e.g., *rework, worker*), and compounds (e.g., *workplace*). Once the reader has learned to recognize a root word or morpheme, the orthographic knowledge of this word or morpheme will facilitate reading words based on the same root like *worker* and *working* (Elbro and Arnbak, 1996).

Several researchers have argued that sensitivity to the language’s morphological structure, in addition to sensitivity to phonemes, plays an important role in the reading process (Elbro and Arnbak, 1996; Casalis and Louis-Alexandre, 2000; for reviews see Mann, 2000, and Nagy et al., 2013), and more particularly in reading difficulties (Ben-Dror et al., 1995; Lyytinen and Lyytinen, 2004; Leikin and Hagit, 2006; Schiff and Raveh, 2007). Morphological awareness has been shown to be correlated with word reading, spelling, and vocabulary knowledge in English and a number of other languages (Kuo and Anderson, 2006; Shu et al., 2006; Schiff and Raveh, 2007). Moreover, a growing body of research demonstrates that, as early as the second grade, developing readers rely on morphemes when processing morphologically complex words and pseudowords, as was shown for English (Carlisle and Stone, 2005), French (Colé et al., 2012), and Italian (Burani et al., 2002). Whereas the importance of phonological awareness, at least in transparent orthographies, has been shown to decrease once the basic decoding rules have been acquired (Holopainen et al., 2001; Leppänen et al., 2006; Georgiou et al., 2008; Vaessen et al., 2010; Furnes and Samuelsson, 2011), the relevance of morphological awareness for reading increases throughout the school years (e.g., Carlisle, 2000; Mahony et al., 2000), and morphological knowledge continues its development across the upper elementary years (Berninger et al., 2010) and beyond (Tyler and Nagy, 1989).

The recognition of familiar morphemes has been found to facilitate accuracy and speed of reading and the spelling of morphologically more complex words (Carlisle and Stone, 2005). Analyzing Hebrew words, Ben-Dror et al. (1995) illustrated that their recognition and understanding could be facilitated by ready knowledge of word structure and rules of word derivation, which may be due to the fact that early knowledge of word structures eases the formation and contributes to the quality of word-specific representations in memory (Acha et al., 2010; Nagy et al., 2013), which in turn facilitates visual word recognition.

Especially in orthographies with an opaque writing system, the morphological structure of words functions like an anchor to the reader (Schiff and Raveh, 2007). Because of their often less transparent grapheme-phoneme correspondences, these orthographies are not only governed by phonology but also by morphology. In fact, many phonemic irregularities may from the morphological perspective be regularities. Silent letters in English, such as in *condemn* and *bomb*, are regular when they occur in *condemnation* and *bombardment.* In addition, the spelling of the phonemically ambiguous letters ‘c’ and ‘s’ in *electricity* and *university*, are spelled in morphological analogy with the words *electric* and *universe* (Elbro and Arnbak, 1996). English is an extreme example of an orthography in which morphological information is also coded in spelling. However, in many other languages the reading process also entails more than a ‘simple’ decoding of grapheme-phoneme correspondences. An illustrative example is French morphology (Carrillo et al., 2013), which is more accurately represented in written than in spoken language since it makes use of numerous orthographic marks lacking any phonological counterparts. This is for instance the case with the final ‘nt’ of verbs and the final ‘s’ in plurals of adjectives and nouns, like the homophones *il chante* (‘he sings’) and *ils chantent* (‘they sing’) where both words are pronounced identically as /il ∫ ᾶt/.

### Morphological Complexity and Reading Acquisition

Although empirical evidence has shown that the time needed to become an accurate and fluent reader is considerably shorter for transparent orthographies with unambiguous grapheme-phoneme correspondences than the period required for orthographies with less consistent and predictable spellings (Seymour et al., 2003), to date little research has focused on whether typological properties, such as morphological complexity of the specific orthography to be acquired, affect the development of visual word-recognition skills and dyslexia.

Stage models of reading such as Ehri’s (2005) suggest that the most proficient readers read multisyllabic words via chunking, which reduces the demand on working memory (Nagy et al., 2013). The word *interesting*, for example, can be read in two chunks via morphemes (*interest + ing)*, sidestepping eleven grapheme-phoneme connections (Nagy et al., 2013). For these long, morphologically complex words, a letter-by-letter decoding strategy would be highly inefficient. However, a major problem with prelexical morphological decomposition is that it cannot differentiate between pseudo-morphemes and real morphemes. Only after the reader has recognized the word, does it become clear whether a particular letter string is indeed the morpheme it resembled. *Car* might, for instance, be the root in *carpet* and *read* in *ready*, even though, of course, they are not.

In the dual-route model of orthographic processing proposed by Grainger and Ziegler (2011), the distinction between the traditional ‘direct’ orthographic and ‘indirect’ phonological routes is expanded with a distinction between two orthographic pathways, most specifically differing with regard to the level of precision with which letter-position information is coded. The authors posit that, with respect to morphological processing, morpho-orthographic decomposition takes place along the *fine-grained orthographic processing route* of their model. A fine-grained orthographic code provides detailed information about the order of the letters in a string necessary for the detection of affixes (e.g., to identify the suffix *-er* in the word *farmer*, or to differentiate between the non-suffix *–oin* and the real suffix *-ion*). The fine-grained route is not limited to the processing of single grapheme representations but is more generally dedicated to optimize processing via the chunking of regularly co-occurring contiguous letter combinations, such as morphemes and multi-letter graphemes. Morphosemantic processing is supposed to occur via a *coarse-grained route* enabling the reader to access morphological representations very rapidly. The coarse-grained code increases the efficiency of the orthography-to-semantics mapping by selecting letter combinations that are the most informative with regard to word identity (diagnosticity) in the absence of precise positional information and independent of the morphological structure of the word. The use of coarse-grained coding for morpho-orthographic decomposition would create too many false affix detections, like detecting the suffix *-er* in *their*, just as would be the case with complex graphemes.

The Finnish orthography provides an interesting example of a language possessing an extremely transparent set of grapheme-phoneme correspondences but a complicated and opaque morphology. The close-to-perfect grapheme-phoneme correspondences and the small number of essential correspondence rules make this writing system optimal for reading acquisition from the phonological decoding perspective. The number of consonant clusters is small and the phonemic structure of syllables simple, allowing the use of left-to-right phonological recoding strategies without the need for explicit graphemic parsing; all factors that should promote the reading acquisition process. When it comes to word-recognition, however, the effectiveness of these ‘beneficial’ factors is reduced by characteristics of the Finnish morphology since the majority of the words are polysyllabic and tend to be long due to the agglutinative morphology, a rich derivational system, and highly productive compounding (Aro, 2004; Lyytinen et al., 2006). Any Finnish noun, for example, can have over 2,000 orthographic forms created by different combinations of plural, case, and a variety of clitics. For verbs, the number of forms is even higher (Niemi et al., 1994). When considering derivations and the highly productive compounding, the number of possible lexical environments in which a Finnish root can exist is enormous (Aro, 2004). Many of the morphological variations of the same words often differ by one phoneme only (e.g., *talo* ‘house’; *talossa* ‘in house’; *talosta* ‘from house’). As a consequence, the reader’s phonological representations must be accurately specified in order to be able to use such inflections (Torppa et al., 2010).

Turkish and Basque are other examples of orthographically transparent languages with an agglutinative morphology (Durgunŏglu and Öney, 1999; Acha et al., 2010). In these languages, syntactic phrases tend to be made up of words formed by stacking functional morphemes to the stem (Acha et al., 2010). In languages in which the morphological structure of a word almost always remains the same regardless of its function in the sentence or the phrase it belongs to, the word will be retrieved with little effort once it has been stored in the orthographic lexicon (Acha et al., 2010). In contrast, the agglutinative nature of some morphological systems results in words of significant length that contain multiple parts of the semantic information. The Finnish word *näytettyämme* (‘after we have shown’) for example, contains the stem *näy*, the derivative *+te*, the past participle *+tty*, case marker *+ä* and the possessive particle *+mme* (Lyytinen et al., 2006). Moreover, given the many root forms that are affected by inflection, the ability to recognize roots does not suffice to recognize words (Aro, 2004).

Lyytinen and Lyytinen (2004) found that by age 3, when Finnish children have developed basic inflectional skills, most of them already have an implicit ability to manipulate small phonological units. More than one third is able to read before the start of formal reading instruction, while more than 95% develops accurate reading skills during the 1st year (Holopainen et al., 2001). To explain the large number of exceptional inflections that are already understood by Finnish children at school-entry age, it has been suggested that these children are highly focused on the details of spoken language in order to differentiate words with small (single phonemic) variations. Such an orientation to small phonemic differences would explain the connection between inflectional morphology and reading accuracy and fluency in Finnish (Torppa et al., 2010). Whereas Finnish children use the grapheme-phoneme correspondences to read Finnish words during their first school year (Holopainen et al., 2002), in the 3rd year they are able to read morphologically complex words better and faster than mono-morphemic words, especially if the words are low-frequency words (Bertram et al., 2000). These findings suggest that children learn to recognize the morphemic structure of Finnish words through the recognition of their constituent stems and morphemes (Acha et al., 2010). When no or only weak full-form representations are available for infrequent mono-morphemic words, little sense can be made of mono-morphemic words that do not contain sublexical units. For derived words, however, sublexical units do exist in the form of rather high-frequent morphemes, and recognizing words based on these units has been shown to offer the young Finnish reader a rather successful back-up option, be it more successful in high- than in low-productive derivations (Bertram et al., 2000).

Consistent with the Finnish findings, there is evidence suggesting that Turkish children are also already skilled at decoding complex pseudowords by the end of the first grade (Öney and Goldman, 1984), while they were also found to develop a sensitivity to word-final elements in their language during the same period: kindergarten and first-grade pupils were shown to be more proficient in deleting final phonemes of words than their English peers (Durgunŏglu and Öney, 1999). Since Turkish is an inflected language, the last part of the word is continuously rearranged when new inflections are added. Variation in suffixes is very common and may result in vowel dropping in suffixes. Being a speaker of Turkish hence requires constant monitoring and manipulation of subword linguistic components, where attention needs to be paid to the phonological characteristics of suffixes and the speaker has to choose between alternate surface forms of the suffix based on phonological criteria (Durgunŏglu and Öney, 1999). Learning to pay special attention to the ending of words could then mediate the progression from decoding to more automatic reading of larger units in Turkish (Acha et al., 2010).

Results from the Acha et al. (2010) study suggest that in Basque visual word recognition is modulated by children’s knowledge of word inflections at the earliest stage of reading. The development of inflected-word recognition was examined in beginning (third-grade), intermediate (sixth-grade), and proficient (student) readers who were all receiving formal instruction in the Basque language. The coexistence of Spanish and Basque in the Basque Country of Spain enabled the authors to compare the reading behaviors of readers with different skill levels in Basque morphology (L1- vs. L2-speakers of Basque). Basque and Spanish both have a transparent and regular orthography. However, whereas Basque is an agglutinative language, Spanish is not. Of particular interest here is that Acha et al. (2010) found that the way in which correctly inflected Basque words were identified across age groups was modulated by the readers’ native language. Third-grade L1-readers of Basque were able to read the correctly inflected words faster than their L1-Spanish peers and were also faster at detecting whether the inflected words were correct or incorrect, showing an error rate that was independent of number and type of inflections, while the L1-Spanish children produced more errors with increasing word length. The authors propose that the L1-Spanish readers have already started storing words but not their inflections, which is why, when inflection decoding was needed, the number of errors increased the more letters words contained. This difference between groups diminished with age. Apparently, as the readers’ vocabularies grow and once stem and inflections have been added to the lexicon, differences at word-identification level between first- and second-language readers tend to vanish. Acha et al. (2010) suggest that in agglutinative languages like Basque, young speakers who are aware of the morphological properties of their native tongue as an intrinsic part of their linguistic knowledge, differ in the way they identify inflected words from same-age second-language learners: the former readers were substantially more proficient and accurate at decomposing and identifying word constituents than their Spanish peers. The authors postulate that in agglutinative languages, young readers use orthographic knowledge they acquire from reading to develop a complete lexical system in which not only words, but also inflectional morphemes are fully represented and retrievable. They additionally propose that, consistent with previous findings for Finnish and Turkish children (Lyytinen et al., 1995; Durgunŏglu and Öney, 1999) young readers of agglutinative languages focus their attention on word endings when searching for salient cues, rapidly discriminating between subtle differences at word boundaries.

### Morphological Complexity and Dyslexia

While the relationship between phonological processing and dyslexia has been extensively studied, much less in known about associations between morphological processing and dyslexia. In their pioneering work, Elbro and Arnbak (1996) presume that dyslexic readers are particularly inclined to rely on morphemes during visual word recognition. They, moreover, may adopt morphological analyses as a compensatory strategy to reduce the negative influence of their phonological deficit on visual word recognition (e.g., Elbro and Arnbak, 1996; Casalis et al., 2004; Leikin and Hagit, 2006). As dyslexic individuals have been shown to have difficulties reading new (Rack et al., 1992) and long words (Martens and De Jong, 2006), visual word recognition may be facilitated by their decomposing morphologically complex words into morpheme-size units (Quémart and Casalis, 2015). While typically developing readers are able to recognize words as a whole by rapidly accessing orthographic and phonological codes, dyslexic readers may be forced to employ morphological decomposition for lexical access as their decoding abilities are weak and whole-word processing would be slow and inefficient (Leikin and Hagit, 2006). Although some researchers argue that processing written morphemes requires the ability to also process small-sized grapheme-phoneme correspondences (e.g., Duncan et al., 2000), others suggest that reading development does not necessarily involve a small-to-large unit progression (Ziegler and Goswami, 2005) and that in dyslexic readers associations between orthography and phonology are made at a coarse-grained level involving multiletter or morphemic units (e.g., Hatcher and Snowling, 2002).

Elbro and Arnbak (1996) propose a meaning-driven hypothesis of morphological decomposition in dyslexic readers (see also Casalis et al., 2004), where the activation of the meaning of morphemes is the central process in morphological decomposition when reading aloud. Supporting their hypothesis and contrary to reading-level controls, the dyslexic children in their study recognized morphologically transparent words such as *sunburn* more successfully than non-transparent items such as *trumpet.* Burani et al. (2008) instead assume that activation of semantic knowledge is not necessarily required in order to process written morphology when reading aloud (see also Traficante et al., 2011). Researchers supporting their form-driven hypothesis of morphological decomposition propose that dyslexic children are better able to capture morphemes than graphemes when reading long and infrequent words because morphemic units are larger than graphemic units and therefore easier to grasp (Quémart and Casalis, 2015).

A number of studies analyzing different languages found dyslexic readers to show greater deficits in morphology than regular readers (e.g., Ben-Dror et al., 1995; Siegel, 2008). Furthermore, Finnish children experiencing difficulties with morphemic identification during the early years of reading acquisition have been shown to have a greater risk of developing dyslexia later on (Lyytinen and Lyytinen, 2004). However, not all researchers agree on whether weak performance on morphological processing tasks is a primary deficit (e.g., Ben-Dror et al., 1995; Joanisse et al., 2000) or a secondary problem caused by a phonological deficit (e.g., Fowler and Liberman, 1995; Shankweiler et al., 1995). Supporters of the first assumption claim that reading disabled individuals lack basic morphological skills due to a delayed language development or deficiencies in the morphological domain itself. This view rests primarily on the finding that phonological and morphological skills have been demonstrated to be relatively independent of each other (Casalis and Louis-Alexandre, 2000; Mahony et al., 2000). Supporters of the latter theory argue that poor performance on morphological tasks largely stems from the same weakness in the phonological component assumed to underlie dyslexia.

Studies exploring the ability of dyslexic readers to make use of morphemes during visual word recognition have yielded inconsistent results. Schiff and Raveh (2007) reported a lack of sensitivity to the morphological structure of words in adult Hebrew readers with dyslexia. They compared the effect of morphological priming in a word-fragment completion task (e.g., *scanner-scan*) with a repetition-priming effect (e.g., *scan-scan)*. Contrary to a strong effect of morphological priming in typical readers, in the dyslexic readers the target completion rate was not influenced. The authors (Raveh and Schiff, 2008) obtained similar results on a primed visual lexical decision task. They (Schiff and Raveh, 2007) suggest that the lack of sensitivity to morphological primes of adult Hebrew dyslexics shows that their lexical access does not involve morphological decomposition. Deacon et al. (2006) found that, in contrast to average adult readers of English, high-functioning dyslexic readers were not influenced by the morphological complexity of words when performing a lexical decision task.

Italian children with dyslexia, on the other hand, have been shown to benefit from the identification of morphemes when reading complex words aloud (Burani et al., 2008; Traficante et al., 2011). Burani et al. (2008), for instance, found an advantage when sixth-grade dyslexic children were asked to read pseudowords composed of morphemes (root + suffix) as compared to pseudowords without morphological structure (non-root + non-suffix). Unlike reading-age peers, dyslexic Danish adolescents were more efficient when they could move a text window morpheme-by-morpheme rather than syllable-by-syllable (Elbro and Arnbak, 1996). Leikin and Hagit (2006) showed morphological priming to facilitate the lexical decision for real words in both dyslexic and regular adult readers of Hebrew. Facilitation effects were similar in form but differed in quantity for the groups, with the dyslexic readers reading significantly more slowly but deriving relatively greater benefit from morphological priming than did the regular readers, suggesting a heightened sensitivity to morphological constituents of words. Note, however, that, regardless of the seemingly regular use of their morphological knowledge, the dyslexic adults scored significantly lower on all morphological awareness tasks.

Quémart and Casalis (2015) reported that, while age-matched and reading-level controls were mostly influenced by the morphemes’ form properties, French dyslexic children relied on morphemes during the visual recognition of complex words which was mainly driven by their semantic properties, confirming the hypothesis of semantically structured morphological representations in dyslexic readers, as proposed by Elbro and Arnbak (1996; see also Castles and Coltheart, 1993). Quémart and Casalis assume that young dyslexic readers rapidly activate the semantic properties of morphemes to try to compensate for their deficit in processing morpho-orthographic information. Following the dual-route model of orthographic processing proposed by Grainger and Ziegler (2011), they argue that the insensitivity of dyslexic readers to small orthographic modifications of the base word underscores the specific involvement of the coarse-grained route during visual word recognition. Given that Grainger and Ziegler (2011) claim this route is selectively involved in morphosemantic processing, Quémart and Casalis’ findings also reinforce the idea that dyslexic readers activate morphosemantic representations only when processing written morphology. Because the orthographic representations of dyslexic readers are insufficiently detailed, priming effects occur even when, orthographically, primes and targets do not perfectly match (also see Marinus and De Jong, 2010). Quémart and Casalis (2015) postulate that in chronological-age-matched and reading-level controls morphological representations are located at the morpho–orthographic level. According to the researchers, the lack of flexibility of their word-recognition system shows that in regular readers orthographic processing is primarily achieved via the fine-grained processing route that is sensitive to form modifications. This processing route is also assumed to be involved in morpho-orthographic decomposition (Grainger and Ziegler, 2011) and confirms earlier findings by Quémart et al. (2011) that showed morphological decomposition to be essentially triggered by the form properties of morphemes in typically developing readers across grades 3 to 7.

Schiff and Raveh’s (2007) findings imply that the word-recognition strategy dyslexic adult readers of Hebrew apply is qualitatively different from procedures used by typical readers, at least at the morphological processing level. This is in sharp contrast to Quémart and Casalis (2015) results that showed that dyslexic French children have developed representations for written morphology and that these representations are activated rapidly and automatically during visual recognition of morphologically complex words. Berthiaume and Daigle (2014) did note some morphological sensitivity among their French dyslexic children aged 9–12 years but also that they were outperformed by both reading-level and same-age peers. The lack of consistency in the results described may be due to methodological differences, such as selected tasks and age or control groups. Nonetheless, it clearly demonstrates the need to further investigate how dyslexic and typical readers process written morphology.

## Syllabic Complexity

Another important issue in understanding visual word recognition is the role the syllable plays. It has been claimed that in reading the lexical processor routinely uses the syllable as a sublexical unit rather than processing the words as a whole (e.g., Taft and Forster, 1976; Prinzmetal et al., 1986). Research has shown that in French the syllable plays an essential role in the perception and segmentation of spoken words, but this is less obvious for spoken English (e.g., Cutler et al., 1986; Bradley et al., 1993).

If a word’s syllabic structure is relevant for word recognition, it becomes necessary to define syllable boundaries for every word. In general, a syllable can be divided into an onset, a nucleus, and a coda, although all languages also feature the simple CV (single consonant and vowel) syllable without a coda (Sprenger-Charolles and Siegel, 1997). The word *script* /skrIpt/, for example, consists of the onset /skr/, the nucleus /I/ and the coda /pt/ (Rouibah and Taft, 2001). A language’s syllabic structure is then defined by a consonant-vowel template (Itô,, 1989) that specifies the maximum number of consonants in onset and coda, as well as vowels in the nucleus. Given this definition of syllabic structure, one should in principle be able to identify an isolated syllable as well as the syllable boundaries within a polysyllabic word (Rouibah and Taft, 2001).

In French, syllable boundaries seem clear-cut, whereas in English they are often less clearly defined (Rouibah and Taft, 2001). The French syllables follow the *maximal onset principle* (Spencer, 1996). According to this principle, a consonant is positioned in such a way that the number of consonant onsets occurring in the word is maximized. In the word ‘routine,’ for example, the consonant /t/ becomes the onset of the second syllable *(rou-tine)* rather than the coda of the first. Taft (1979) claimed that English-speaking readers can segment words according to orthographic sublexical units that do not necessarily correspond to phonological syllabic units. He proposed the idea of a unit that maximizes the coda of the first syllable by drawing the structural boundary after all the consonants that follow the first vowel of the stem morpheme (e.g., *murd-er* or *sir-en*), calling this initial unit the *Basic Orthographic Syllabic Structure* (BOSS). Cutler et al. (1986) argue that the aforementioned finding that French native speakers use a syllabification strategy to segment spoken words whereas English speakers do not, may be attributable to the French language having ‘easy-to-syllabify’ words while syllabification is more difficult in English words.

Various definitions are proposed to describe the concept of syllabic complexity. Fenk-Oczlon and Fenk (2008), for example, define it as the number of phonemes per syllable. A broader definition is introduced by Adsett and Marchand (2010), who define syllabic complexity as a measure of how difficult it is, on average, to determine the syllable boundaries in words in a specific language. Seymour et al. (2003) used syllabic complexity, in addition to orthographic depth, to describe the level of orthographic complexity in the alphabetic writing systems included in their sample (COST Action A8; Niessen et al., 2000). According to Seymour et al. (2003), the syllabic-complexity dimension differentiates between Germanic and Romance languages. Germanic languages are characterized by closed CVC syllables and complex consonant clusters in both onset and coda positions (e.g., Danish, German, and English). Research has shown that the spelling of consonant clusters poses a major phonological problem to young learners; clusters are treated as phonological units and are difficult to split into their separate phonemes (Treiman, 1991). These difficulties young learners experience might reflect a general deficit in phonological segmentation and in identifying phonemes in spoken syllables. Moreover, the high level of co-articulation in the consonant morphemes in the cluster might negatively influence the process (Serrano and Defior, 2012).

In contrast to Germanic languages, the Romance type languages have a predominance of open CV syllables with few initial or final consonant clusters (e.g., French, Spanish, and Italian). Seymour and colleagues postulated that the effort required to acquire literacy increases from shallow to deep orthographies and from simple to complex syllable structures. In other words, the deeper the orthography and the more complex the syllable structure, the more complex the orthography can be expected to be. If orthographic complexity impacts the foundation phases of reading acquisition, they posit that the initial steps in reading would be mastered more quickly in orthographies with simple syllabic structures than they would in those with complex syllabic structures, and that acquisition would be drawn-out in deeper orthographies than in shallow orthographies.

### Syllabic Complexity and Reading and Spelling Acquisition

Several studies support the hypothesis that specifically consonant clusters may pose an additional problem to the young learner. In English, very young children were found to have difficulty pronouncing initial consonant clusters (Treiman and Weatherston, 1992). Read (1975) discovered an interesting phenomenon among preschoolers beginning to spell unaided, noticing that children sometimes failed to spell the nasals /n/, /m/, and /upeta/ when they occurred before another consonant. The words *went* and *and* were thus misspelled as ‘wet’ and ‘ad.’ Nasals in other contexts, such as at the beginnings of words, were rarely omitted. These errors are indicative of a general deficiency in capturing the internal structure of clusters in spoken words. As young spellers of English have been shown to have problems with both initial and final consonant clusters, it is suggested that this reflects a more general deficit in segmenting syllables that contain clusters (Treiman, 1991). As a result, some children fail to spell the word with the appropriate letters.

The nature of this ‘cluster’ problem differs for initial and final clusters. With final consonant clusters, young children pair the cluster’s first consonant with the preceding vowel, at least when the consonant is a nasal (Read, 1975). If the /n/ in the word *sand*, /sænd/, is considered to form a unit with the /æ/, children may be more likely to drop the ‘n’ than the ‘d’ in their spelling (Treiman, 1991). Indeed, research has shown that young spellers are more prone to omit the first phoneme of a final cluster than the second (Read, 1975; Marcel, 1980; Treiman, 1993).

With initial clusters, children have been shown to group the second consonant of the cluster with the first, treating the two consonants of the cluster as a unit, the syllable onset. As a result, they may sometimes spell the onset with the single letter rather than with a cluster (Marcel, 1980; Bruck and Treiman, 1990; Treiman, 1991; Sprenger-Charolles and Siegel, 1997). Marcel (1980) revealed that some 8- and 9-year-old children who lagged at least 1 year behind their peers in reading and spelling, made errors such as ‘tay’ for *tray*. Bruck and Treiman (1990) found similar errors among dyslexic children and, to a lesser extent, among typical first- and second-grade readers. Both groups had problems spelling syllables with initial consonant clusters, sometimes failing to represent the cluster’s second consonant. Treiman’s (1991) study demonstrated that kindergarten and first-grade children tended to make spelling errors that concerned the simplification of complex onsets, omitting the consonant in second position, whether it was in the initial syllable or not. The initial consonant of these complex onsets was less likely to be omitted. When they were asked to name pictures and to state whether the name contained a specific target letter, the children identified the consonant less often when it was the second element in a consonant cluster than when the same consonant did not belong to a cluster. These problems were shown to be transient in nature, however, as the children had fewer second-consonant omissions during the second than during the first half of the school year. A study by Steffler et al. (1998) similarly revealed that second-grade children made more spelling errors on CCVC words than higher-grade children. Yet, the awareness that in words such as *play*, the onset /pl/ contains two parts appears to come slowly for some children. In Treiman’s (1991) study, onset clusters continued to cause some children difficulty up through third grade. Serrano and Defior (2012) did not find any such performance differences in typical readers reading simple items and items with consonant clusters. However, the children in their sample were older (aged 9–16) than in Treiman’s (1991) and Steffler et al. (1998) studies.

Besides in English, consonant clusters have been shown to cause problems to the young learner in other languages as well. Schreuder and Van Bon (1989) demonstrated that isolating initial phonemes in Dutch was more difficult in consonant clusters. In their study on the relationship between phonemic segmentation performance and reading and writing ability, they observed that for first-grade children it was not only difficult to segment consonant clusters but that it also had an adverse effect on their processing of segments earlier on in the word. Spanish studies (Defior et al., 2003, cited by Jiménez González and Jiménez Rodríguez, 1999; Serrano and Defior, 2012, cited by Serrano and Defior, 2012), moreover, showed that children experienced difficulties in spelling consonant clusters in the early stages of writing acquisition.

Sprenger-Charolles and Siegel (1997) conducted a longitudinal study of the effects of syllabic structure on reading and spelling development in French using both bi- and trisyllabic pseudowords. They found the first-graders to have more difficulties reading and spelling items with more complex syllabic structures including CCV and/or CVC syllables than those consisting of simple CV syllables. Almost all cases of deletion errors involved the deletion of a coda or the simplification of a complex onset, reducing the syllable to its primary elements C+V. As Marcel (1980) and Bruck and Treiman (1990) had reported previously, it usually was the second element in the complex onset that was omitted, while in reading this typically were the codas at the end of pseudowords. Sprenger-Charolles and Siegel (1997) furthermore showed that, consistent with the Hierarchy of Sonority (Clements, 1990), the phonological properties of consonants (sonority) explained the majority of deletions and not the position within the syllable or word. The most sonorant consonants were most likely to be deleted because sonorant consonants are phonologically the closest to the vowel (e.g., more deletions of liquids than obstruents in clusters). They also demonstrated that not the visual characteristics, but the principal phonological categories were preserved in substitutions; consonants were replaced by consonants, and vowels by vowels belonging to the same phonological category (e.g., liquids for liquids, fricatives for fricatives, and stops for stops). Contrary to their expectations, the relative frequency of open syllables (CV) in French, as opposed to the number of closed syllables (e.g., Delattre, 1965), did not result in greater performance on (open) CV and CCV syllables compared to (closed) CVC syllables.

Lee and Wheldall (2011) investigated letter knowledge, phonological awareness, and word reading in 46 first-grade Malaysian children while additionally charting the children’s reading performance of words with different syllable structures. In the Malay language, the syllable is a salient unit as most words are bi- and multisyllabic. Moreover, the language has a simple syllable structure and clear syllable boundaries (Winskel and Widjaja, 2007). The children evidently found words with a simple open CV syllable structure easier to decode than words with diphthongs, digraphs, or words containing the vowel ‘e.’ Here, it is worth mentioning that Malaysian has a highly transparent writing system with a close-to-perfect grapheme-phoneme correspondence. The grapheme ‘e’ is an exception, because it has two phonemic forms (i.e., /e/ and /?/). As the complexity of the syllabic structure increased, a corresponding decline in performance occurred. Besides the presence of certain more complex graphemes, the position of graphemes in a word were also found to affect word-recognition. Words with a digraph at the beginning (e.g., *syarikat*, ‘company’) were more difficult to decode than words with a digraph at the end of the word (e.g., *batang*, ‘shaft’). Furthermore, words with two vowel graphemes belonging to different syllables occurring together in the middle of a word (e.g., *soal*, ‘about’) or at the end of a word (e.g., *tua*, ‘old’) proved problematic to the new readers due to confusion over the location of the syllable boundary, while, finally, shorter stem words proved easier to read than longer multisyllabic words with derivational affixes.

Seymour et al. (2003) study demonstrated that syllabic complexity selectively affected the decoding of pseudowords, whereas orthographic depth affected both word and pseudoword reading. Pseudoword reading abilities of first- and second-graders were shown to significantly differ for each native language evaluated, with differences coinciding with the complexity of its syllabic structure. The authors emphasized that the syllabic-complexity effect was evident when simple pseudowords were read, based on single letter grapheme-phoneme correspondences in the absence of any consonant clusters or multi-letter graphemes. They concluded that straightforward letter-sound decoding is more difficult to acquire in languages with complex phonologies than it is in languages with a simple phonology and venture that the embedding of grapheme-phoneme correspondences in consonant clusters may impede the learning process. Thus, correspondences between the grapheme ‘p’ and phoneme /p/ in English, for example, occur in isolation but also in various consonant clusters such as ‘*sp*,’ ‘*spr*,’ and ‘*mple.*’ Arguably, in languages with a greater syllabic complexity, material for new readers will necessarily require more skill in recognizing such correspondences in clusters, slowing down the learning process.

### Syllabic Complexity and Dyslexia

In their study on the spelling abilities of 31 Spanish dyslexics whose ages ranged from 9 to 16 years, Serrano and Defior (2012) showed that the spelling of consonant clusters presented the dyslexic students with more difficulties than it did typically developing readers who were matched for reading level and chronological age. In spite of its simple linguistic structure and the predominance of open syllable structures (CV), the Spanish orthographic code incorporates certain complexities that affect literacy acquisition and learning problems such as developmental dyslexia, among which its consonant clusters. They found the performance difference for simple items and items with consonant clusters to be greater among dyslexic readers; the dyslexic readers consistently performed poorer on items with consonant clusters, while the overall performance of the typical readers was similar for both syllable-structure types.

The study by Bruck and Treiman (1990) we discussed earlier had revealed that English dyslexic readers aged 7 to 13 years made more errors in the auditory recognition of target phonemes than did typical reading-level-matched controls, but here the authors found a similar pattern of errors for the two groups, where pseudowords containing consonant clusters proved especially problematic.

Struiksma (2003) showed that fourth-grade children with low reading skills in Dutch continued to have difficulties reading words containing consonant clusters despite intensive and focused instruction. The problems were most apparent when the children were asked to read words beginning with the same consonant clusters that were followed by different vowels (e.g., blaf – blik – blok, ‘bark’ – ‘can’ – ‘block’). Switching between the vowels in CCV sequences complicated the reading process significantly, possibly due to co-articulation whereby the articulation of one speech segment is being influenced by the articulation of another (Snellings et al., 2010) requiring stop consonants to be identified based on neighboring vowel transitions (e.g., Pols and Schouten, 1978). Despite the fact that in Struiksma’s study the spelling of the consonant-cluster sounds was identical, their acoustic properties changed with each different vowel, which increased task demands.

Based on Treiman’s (1991) and Struiksma’s (2003) findings, Snellings et al. (2010) predicted that the perception of consonants in clusters would be more difficult for Dutch dyslexic children in grade 2 than the perception of single consonants, leading to more errors and prolonging processing time. In their study, pairs of pseudowords were orally presented and the children were asked to decide as fast and as accurately as possible whether the stimuli within each pair were identical or different (e.g., /prar/ – /trar/, or /pa/ – /pa/). The results showed that discriminating clusters was not more difficult than discriminating single consonants and processing times were similar, which led Snelling et al. (2010) to conclude that, apparently, straightforward recognition whether sounds were identical or different was not hindered by surrounding clusters. Unlike Treiman (1991), who demonstrated that consonants in second position of an initial consonant cluster were especially problematic, Snellings et al. (2010) found no differences in the proportions of correct detections between those consonants and single consonants. Snellings et al. (2010) asserted that the children evidently knew which component phonemes were in the second position within the cluster, indicating that in Dutch a lack of phoneme awareness was not the cause of the problems with consonant clusters that Struiksma (2003) had reported. Landerl and Wimmer (2000) showed that phoneme segmentation of onset consonants also posed no problem to German dyslexic third-grade students either, which coincides with Vellutino et al. (2004) later claim that in shallow orthographies phoneme awareness may be less problematic for reading-disabled children (but see Patel et al., 2004, for a different view).

## Discussion

Reading is a complex activity requiring the processing of graphic information in order to achieve optimal text comprehension (Berthiaume and Daigle, 2014). The simple view of reading, a model suggested by Gough and Tunmer (1986) and Hoover and Gough (1990), in short holds that reading comprehension skills can be predicted from two components: decoding abilities, defined as efficient visual word recognition, and linguistic comprehension, which is the ability to use information at the lexical or word level to achieve sentence and discourse interpretations (Hoover and Gough, 1990). Both components are considered necessary for reading success, while neither decoding capacity nor linguistic comprehension by itself is sufficient. Taking this into account, in our review we nevertheless exclusively focused on decoding and visual word recognition.

Learning to read is accomplished earlier in some orthographies than others. Theoretical concepts (i.e., the ODH, Katz and Frost, 1992; the grain size theory, Ziegler and Goswami, 2005) as well as empirical evidence (Aro and Wimmer, 2003; Seymour et al., 2003) suggest that transparent writing systems with highly consistent letter-sound correspondences are acquired more easily than complex and opaque orthographies containing a large number of irregular and inconsistent spellings. Moreover, the level of orthographic transparency has been shown to influence the expression of dyslexia.

However, there is more to reading than grapheme-phoneme consistency, just like there is more than one way in which the reader can segment a written word. Besides dividing words into the graphemes linked to a specific sound, one can segment words based on syllables, resulting in larger phonological units than phonemes. It is widely accepted that visual word recognition is mainly founded on these two types of phonological processes and a great number of studies have accordingly investigated phonological awareness and abilities in dyslexic readers (e.g., De Jong and Van der Leij, 2003; Ziegler and Goswami, 2005; Sprenger-Charolles et al., 2011). Yet another important aspect of word segmentation involves morphological processing, which is the focus of an increasing number of studies (e.g., Mahony et al., 2000; Schiff and Raveh, 2007). Several papers also included analyses of morphological processing in struggling readers such as dyslexics (e.g., Casalis et al., 2004; Burani et al., 2008; Siegel, 2008) and suggest that a deficient development of some of the processes related to word recognition can partially explain the reading difficulties of dyslexic readers (Berthiaume and Daigle, 2014). Although it is unlikely that dyslexic readers can become skilled readers without developing efficient phonological processing skills, they may compensate for their phonological deficit during visual word recognition by processing at the morpheme level (e.g., Elbro and Arnbak, 1996; Casalis et al., 2004; Leikin and Hagit, 2006).

Comparisons across orthographies have prompted several hypotheses aiming to explain how the word-reading process is affected by orthography-specific variations. The way in which phonological, orthographic, and morphological processes function, is shaped by the specific orthography being used, necessitating orthographic-specific strategies when learning to read. According to the universal phonological principle (UPP) as proposed by Perfetti et al. (1992), specific mapping differences across orthographies produce differences in the units of language that are activated in the earliest stages of reading as part of a universal dependence on spoken language and universal involvement of phonology. Whereas the Chinese writing system maps graphs to syllabic morphemes, in alphabetic scripts, graphs are mapped to phonemes. The UPP thus unites the Chinese and alphabetic writing systems at the functional principle level but acknowledges important differences emerging at more detailed levels. In Chinese, phonology is not represented at the phoneme level and its representation at syllable level has been suggested to discourage its use (Perfetti and Harris, 2013). Following the ‘threshold-cascade’ distinction originally introduced by Coltheart et al. (1993) and Perfetti et al. (2005); Perfetti and Harris (2013) argue that Chinese characters are processed threshold-style during reading, in contrast to cascade-style recognition of words in alphabetic orthographies. These styles differ in relation to the moment of activation of phonology relative to orthography. During alphabetic cascaded visual word recognition, the activation of phonemes occurs with grapheme activation based on established correspondences between them. This phonological activation can take place prior to the moment of word identification. Perfetti and Harris (2013) postulate that this processing style is not possible in Chinese, in which phonology is activated only once an orthographic recognition threshold has been reached. During threshold-style processing, immediate activation of the corresponding syllable takes place as the Chinese characters are recognized, allowing the activation of meaning directly by the character. The authors argue that, due to the high level of homophony in Chinese, a process that connects a character only with its meaning appears to be ‘encouraged.’ Thus, the reading procedures that are being developed adapt to the demands of the writing system through the specialization of brain networks that support efficient word identification. This specialization increases with further reading development, leading to differences in the brain networks for alphabetic and Chinese reading (Perfetti and Harris, 2013) as well as to different activation patterns for reading in, for example, English compared to the transparent Italian orthography (Paulesu et al., 2000). It is these specific and vital adjustments made to the reading process depending on the orthography used that have implications for new readers and the development of reading difficulties like dyslexia.

Our review of the current literature on orthographic transparency and syllabic and morphological complexity of alphabetic languages served to gain a better understanding of the orthographic components that influence reading acquisition and dyslexia across the languages researched. Many examples of effects on reading acquisition and the ‘developmental footprint’ (Ziegler and Goswami, 2005) were given. For future research, we would suggest that more cross-linguistic studies be conducted comparing two orthographies which are similar on as many aspects as possible, but different with regard to orthographic transparency, syllabic complexity, or morphological complexity. The quantitative indices discussed in Borleffs et al. (2017) to measure differences between alphabetic languages may provide a starting point to compare languages on the specific aspect studied. Moreover, the proposed results will need to be corroborated by behavioral data of reading acquisition and skilled reading to validate their value in the study of reading development and dyslexia. In addition to behavioral research, there is need for more in depth research to systematically investigate the procedures by which the human brain retrieves linguistic meaning from written texts in different orthographies. The ultimate goal would be to use our in-depth knowledge of the language-specific adjustments needed for the development of efficient reading skills to devise language-specific instruction and interventions (Lyytinen et al., 2015) that address the potential pitfalls resulting from the particular characteristics of the orthography being acquired.

In their attempt to devise a language-specific intervention for English in the United Kingdom, Kyle et al. (2013) assessed the effectiveness of the so-called ‘GraphoGame’ (GG) method among 6- and 7-year-old pupils whom their teachers had identified as having relatively poor reading abilities. The two computer-assisted reading interventions built on research showing that interventions that combine training in phonological skills with explicit training on the correspondences between graphemes and phonemes are the most effective for young speakers of English (e.g., Torgesen et al., 1999; Hatcher et al., 2006). The first game (‘GG Phoneme’) focused specifically on phoneme-level connections between letters and sounds, incorporating theoretical views on the relevance of a small-unit approach to literacy instruction, even for non-transparent languages such as English (e.g., Hulme et al., 2002; Johnston and Watson, 2004). The second game (‘GG Rime’) introduced and reinforced grapheme-phoneme correspondences via rhyming-word families, explicitly focusing on orthographic rime units and demonstrating how rime units and grapheme-phoneme correspondences are related in English spelling. GG Rime was based on the notion that English-speaking pupils will benefit from a focus on oral rhyme and rhyme analogies as part of reading instruction (Goswami and Bryant, 1990). The rhyme-analogy approach proposes that young readers infer connections between their phonological knowledge and the orthography they are trying to master and that in English some of these connections are at the psycholinguistic grain size of rime (Kyle et al., 2013). During learning, they may then develop multiple recoding strategies to enable them to decode English words at more than one grain size. Moreover, in order to develop fully specified orthographic representations of words, all possible grain sizes in phonology and orthography need to be connected (Ziegler and Goswami, 2005).

Kyle et al.’s (2013) two intervention games were presented during 2nd-year reading instruction, supplementing the ongoing classroom literacy instructions. In comparison with the results obtained in untreated controls, the effect size data showed that both interventions had led to considerable gains in reading, spelling, and phonological skills, where the gains in reading achieved with GG Rime were similar or superior to those achieved with GG Phoneme. Comparing effect sizes, improvements in phonological awareness attained with GG Rime were large at both the phoneme and the rhyme level, whereas improvements following GG Phoneme were large at the phoneme but small at the rhyme level. These findings are in line with the training outcomes described by Goswami and East (2000) and Hatcher et al. (2004). Goswami and East (2000) showed, for example, that rime-based literacy instruction led to improvements on measures of phonological awareness at large grain sizes in 5-year-old beginning readers, whereas age peers who had been training grapheme-phoneme correspondence skills only showed relatively poor large-unit awareness.

Kyle et al. (2013) argue that interventions such as theirs seem to have great utility in supporting reading instruction in non-transparent orthographies such as English. By its special focus on rime, GG Rime supported the children’s learning by accessing psycholinguistic units that are already well-developed in young children prior to reading instruction (Goswami, 1999) and that, at least in English, tend to be more consistent than smaller grain sizes such as phonemes (Treiman et al., 1995). During reading acquisition, (remedial) instruction exploiting rime may then serve both the demands and nature of the literacy tasks and the characteristics of the English orthography.

The studies we have reviewed in this paper have deepened our understanding of how reading acquisition and dyslexia are influenced by the linguistic properties of the specific orthography being learned. With our review we also sought to direct attention to areas to which future research could contribute, most specifically in the domain of orthographic structures and literacy acquisition and to the development of effective instruction and intervention methods. Despite our growing insight, we still need to learn more about techniques that teach struggling readers how to make use of language-specific orthographic, morphological, or syllabic resources.

## Author Contributions

EB is the main author of this work and has put most effort in writing, revising and researching the paper’s content. FZ and BM have made substantial contributions throughout the process of selecting and interpreting papers included in this review, and have played a critical role in drafting and revising the content. HL has made substantial contributions to the paper by providing critical feedback on the content and by suggesting ways to further improve the paper. All authors have approved the version to be published.

## Conflict of Interest Statement

The authors declare that the research was conducted in the absence of any commercial or financial relationships that could be construed as a potential conflict of interest.

## Footnotes

1 *Rime* is the intrasyllabic unit that consists of a nucleus and a coda which is optional, e.g., /æt/ in ‘*at,’* ‘*sat,’* and ‘*flat.’* Words that end in the same rime will *rhyme.*
